# Consumption of a diet high in fat and sugar is associated with worse spatial navigation ability in a virtual environment

**DOI:** 10.1038/s41366-025-01776-8

**Published:** 2025-04-17

**Authors:** Dominic M. D. Tran, Kit S. Double, Ian N. Johnston, R. Frederick Westbrook, Irina M. Harris

**Affiliations:** 1 School of Psychology, The University of Sydney, Sydney, NSW Australia; 2 School of Psychology, UNSW Sydney, Sydney, NSW Australia

**Keywords:** Cognitive neuroscience, Feeding behaviour

## Abstract

**Background:**

The Western diet is rich in saturated fats and refined sugars. Overconsumption of this diet can lead to obesity, metabolic and cardiovascular disease, as well as certain types of cancers. Evidence suggests that this diet also has adverse effects on cognitive function. Regular consumption of fats and sugars is associated with faster rates of age-related cognitive decline in middle age and older adults. Experimental studies using rodent models show that diets high in fats and sugars can impair brain functions, particularly in the hippocampus, affecting spatial learning and memory.

**Methods:**

The current study tested the relationship between diet and spatial navigation ability in people using a virtual reality maze. Accurate performance in the maze requires participants to estimate distance and direction information to track self-referential positioning and remember landmark locations.

**Results:**

We found that young adults who frequently consumed foods high in fat and sugar were worse at remembering the location of a treasure chest in the virtual maze. Critically, this relationship remained after controlling for body mass index and performance on a non-spatial task.

**Conclusions:**

The results highlight the impact of diet beyond traditional indicators of physical health, and reveal the specificity of the association between diet and spatial ability. These findings are consistent with those from animal studies and are the first to reveal the adverse effect of diet on spatial learning and memory in a task that requires navigation in three-dimensional space. The results confirm the importance of making healthy dietary choices for cognitive health.

## Introduction

The diet eaten by many people in developed countries is rich in saturated fats and refined carbohydrates. It has long been recognized that excessive intake of this so-called Western style diet results in increased body weight, a progression into obesity, the development of metabolic and cardiovascular disorders [1], as well as some forms of cancer [2]. Evidence has also begun to link intake of this diet with cognitive impairments. For example, epidemiological studies have shown that intake of foods high in fat and sugar by middle-aged and older adults predicts faster rates of normal age-related cognitive decline and increased risk of neurological disorders [e.g. [3, 4]], Moreover, the deleterious effects of this diet on cognition are not confined to the middle-aged and elderly. Epidemiological studies have found prospective and correlational evidence of diet-induced cognitive impairments across childhood and adolescence [5, 6].

Rodent studies have confirmed that consumption of foods that are high in fat and sugar (HFHS) leads to impairments in cognition, especially on tasks requiring the hippocampus and surrounding cortices [7–12]. These studies have also shown that cognitive deficits occur rapidly, well in advance of diet-induced increases in body weight and associated metabolic changes. For example, Tran and Westbrook [13, 14] found that rats fed a Western style, HFHS diet for as little as one week exhibited deficits in a hippocampal-dependent place recognition memory task but not in a cortically-dependent object recognition task. Importantly, Tran and Westbrook [15] also showed that the place recognition deficit involved an inability to use distance and direction information (i.e., spatial geometry) to remember the location of objects. The authors did so by exploiting the well-documented finding that rats and other vertebrates, including young children, typically favor geometrical over featural cues for orientation in rectangular environments with distinctive features and patterns on the walls [16, 17]. They found that rats fed standard chow used geometric information to encode object location in such arenas; the rats showed spatial confusion for an object moved to a geometrically symmetrical (opposite) corner in both featureless and cued arenas. HFHS rats also showed spatial confusion in a featureless arena, which had nothing to distinguish the diagonally opposite corners, but showed *good* recognition memory in the cued arena. The results were taken to mean that HFHS rats used features and patterns to disambiguate the corner location in the cued arena because the default geometry system was impaired. In contrast, control rats relied on geometry over features and were confused by the symmetrical rotation, despite having distinct features to distinguish the corners.

The nature of diet-induced cognitive impairments in people is much less well understood. However, a recent meta-analysis of human studies has reported that, as in rodents, HFHS diets selectively impair hippocampal-dependent forms of cognition, such as spatial navigation [18]. This evidence raises the possibility that, as in rats, HFHS diets in humans would impair the use of geometrical information in spatial navigation tasks. The present experiment examined this possibility. To do so, we developed a virtual navigation task to assess how people incorporate distance and directional information from landmarks into a cognitive map. We hypothesized that consumption of a HFHS diet would be negatively related to the use of distance and directional information.

## Method

### Participants

One hundred and twenty students at the University of Sydney participated in exchange for credit in a Psychology course. Given the study was an individual differences design involving one-on-one testing and a substantial dropout rate was anticipated, we aimed for 100+ participants and recruited as many participants as possible within budgeting and practical constraints. All methods were performed in accordance with the protocols approved by The University of Sydney Psychology Low Risk Ethics Committee (document no. 2017/645), and informed consent was obtained from all participants at the start of the experiment. Sixty-five participants did not complete the task due to either motion sickness induced by the virtual environment (*n* = 47), running out of time and having incomplete or missing data (*n* = 11), technical problems with the equipment or computer program (*n* = 4), or failing to follow task instructions (*n* = 3). The motion sickness or cybersickness dropout rate was thus approximately 39%. Cybersickness rates are poorly reported in the literature but can vary between 12 and 50% [see also [19, 20]]. Depending on the nature of the VR task, cybersickness has been estimated to be greater than 20–40% [21]. Of the 58 participants who experienced motion sickness or had missing data, 46 were well enough to continue and complete (at least some of) the computer tasks. The summary statistics of participants who did and those who did not complete the VR task are reported in Supplementary Table 1. Participants who experienced cybersickness or had incomplete data tended to be female, but did not differ on the dietary or cognitive measures. The remaining participants (*N* = 55) had a mean age of 20 years (SD = 3.2), mean height = 172.8 cm (SD = 9.6), and mean weight = 69.2 kg (SD = 9.6). The female participants (*n* = 24) had a mean height = 166.3 cm (SD = 7.5), and mean weight = 61.7 (SD = 13.6); the male participants (*n* = 30) had a mean height = 178.3 (SD = 7.7), and mean weight = 75.1 (SD = 11.0); one participant preferred not to report their gender.

### Materials

#### The virtual environment

The virtual environment was programmed in Unity 3D software (Unity Technologies, Version 5.3.1) and presented stereoscopically via a head-mounted virtual reality display. An Oculus Rift Development Kit 2 (Oculus VR, Irvine, CA) tracked head movements which were used to determine the view and orientation of the virtual environment. The Oculus display was updated at a rate of 1000 Hz. Each participant controlled their virtual position with a joystick on a Bluetooth controller (Samsung El-GP20HNBEGWW Gamepad). The facing direction was used as the reference point for movement through the environments, such that tilting the joystick up moved the participant forward. Participants were required to stand for the duration of the experiment.

**Fig. 1:**
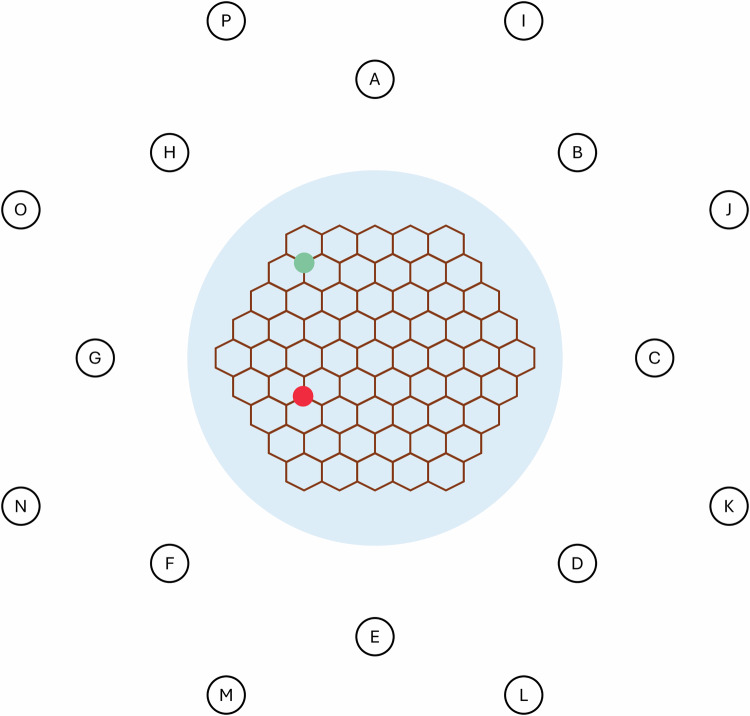
A schematic top-down view of the virtual reality maze. The maze is surrounded by water and two concentric rings of landmarks **A–P**. The green/top dot indicates the start location, and the red/bottom dot indicates the chest location. Landmarks were a mixture of 3D models from the Unity Asset Library, such as buildings, statues, rockets, vehicles, and terrain.

The virtual environment was based on the water maze [22], widely used to study spatial navigation in rodents. It consisted of a virtual pool bounded by a grass terrain and surrounded by two concentric rings with eight landmarks in each ring (see Fig. 1). The landmarks served as location cues that participants could use to orient and position themselves within the maze. There was a static sky above the terrain with the sun located in the NE quadrant and clouds scattered in fixed positions around the sky. To constrain navigation within the pool and create the opportunity for participants to navigate without using the landmarks, a plank maze in the shape of a hexagonal grid was located above the pool. Therefore, spatial position could be encoded and used in two ways: via distance and direction relative to landmarks around the pool, and via distance and direction relative to the start position on the planks.

**Fig. 2:**
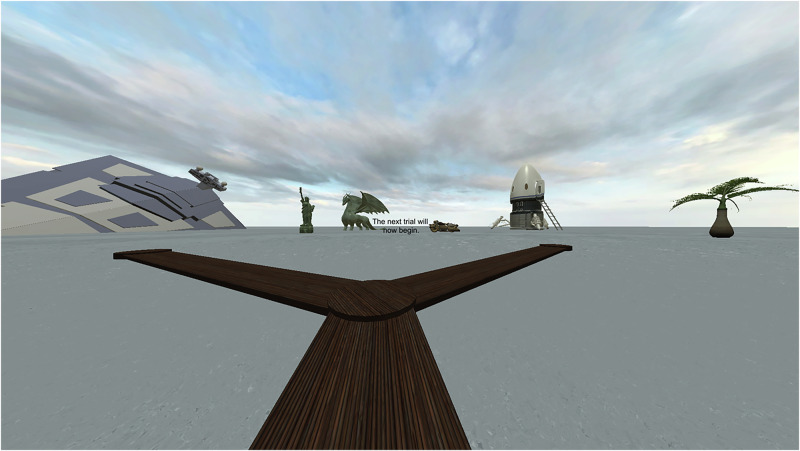
View of the training maze including landmarks in the background and the planks that are currently visible. Text: “The next trial will now begin”. Note that the VR environment used in the experiment differed slightly in the scene textures. This scene is taken from a similar, non-VR version of the task.

To prevent participants from using the hexagonal grid as a visual cue for orienting themselves, only the planks connected to the intersection last visited by the participant were visible. Planks also disappeared when participants left the intersection such that no more than three planks were visible at any given time (Fig. 2).

#### Dietary measure

Participants completed the Dietary Fat and Free Sugar-Short [23] that was designed to estimate the average intake of saturated fats and refined carbohydrates. It asks participants to report the approximate frequency of their consumption of 25 different high fat and high sugar foods over the past 12 months on a five-point categorical rating scale that ranged from ‘1 per month or less’ up to ‘5+ per week’. The specific wording of the question was: “Think about the food you’ve eaten over the past year. Remember breakfast, lunch, dinner and eating out”. The final question asks participants to indicate the number of teaspoons of sugar added to beverages, cereals, or food per week, rated on a scale from 1 to 5, with 1 being “none” and 5 being “7 + ” teaspoons. The DFS was also designed to identify respondents high in either key nutrient alone, or both together. Thus, in addition to the total score, items on the DFS fall into three subscales, those high in fat but not sugar (DFS fat), high in sugar but not fat (DFS sugar), or high in both fat and sugar (DFS fat and sugar).

#### Cognitive measure

A backward digit span task [24] was used to provide a measure of working memory that should be independent of spatial cognition. The task was implemented in Inquisit. Participants were shown a number span and asked to recall the digits in the reverse order of that presented. The task consisted of 14 trials which began with a span of two digits and increased by one with each correctly recalled span or remained at the same span when an error occurred. If two consecutive errors were made, the span decreased by one on the next trial. Participants were required to pass a practice phase of between two and eight trials in order to complete the task. Working memory was operationalized as the two-error maximum length, the last correct digit span before two consecutive errors.

### Procedure

The study was conducted in a quiet room. After a brief demonstration of the VR controller (left-hand only), participants were fitted with the Oculus Rift headset. On the training trials, participants were required to navigate through the environment from a starting location to a target location that contained a treasure chest. Both locations remained fixed for the entirety of the training phase (6 trials). The start location was in the NW quadrant and the location of the target was in the SW quadrant. If participants found the target in less than four minutes, they continued to the next trial. If participants failed to find the target in this time, they were teleported to the target location and given 10 s to familiarize themselves with the location before the next trial started. During these 10 s, they could turn 360 degrees on the spot to observe the relative position of the landmarks but could not move through the maze. Training trials recommenced when the participant indicated they were ready or after 15 s elapsed.

The target was located at a plank intersection and a treasure chest appeared when participants reached that intersection. Participants were told that the appearance of the chest indicated that they had reached the target location. The chest did not appear until the participant had traversed more than halfway down one of the planks leading to the intersection. The appearance of the chest was accompanied by a brief auditory cue. The end of an unsuccessful trial was accompanied by a different auditory cue and participants were then teleported to the target location. Teleportation was instantaneous. Consequently, participants were unable to view any potential paths between their last location and the target, but were able to learn where the target was located relative to the surrounding landmarks.

**Fig. 3:**
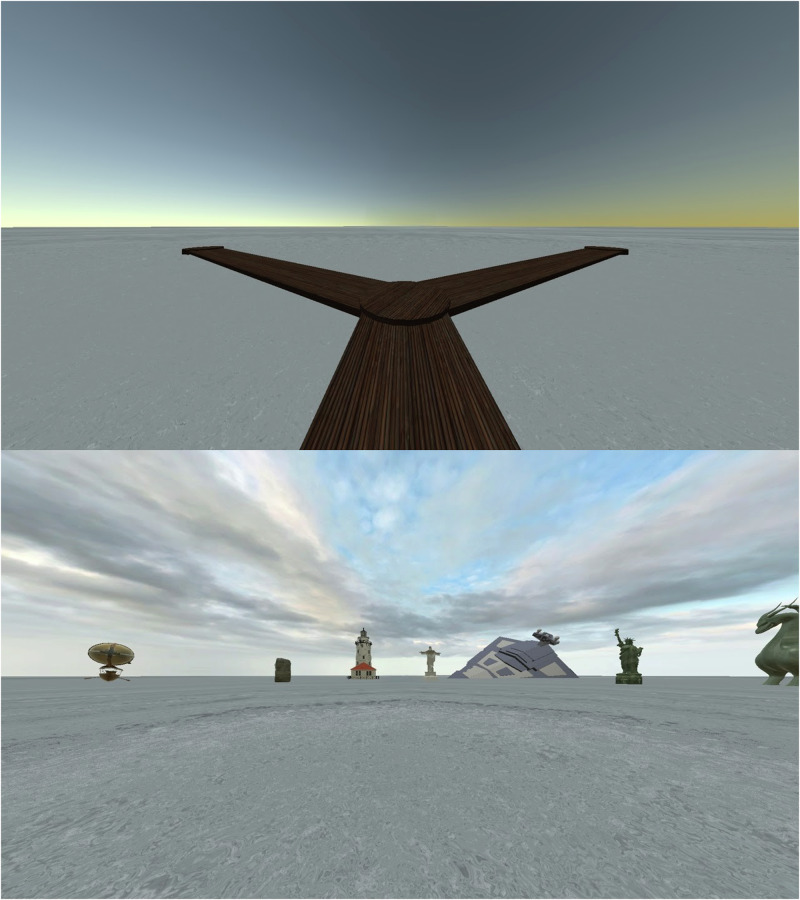
View of the test trials. Top: egocentric navigation using only the planks. Bottom: allocentric navigation using only the landmarks. Note the technical issue with the egocentric navigation described in the text; data are only reported from the allocentric navigation.

Two types of test trials followed the training phase. In both, participants were told that the treasure chest would no longer appear but that they had to navigate from the start location to the target location. Test trials were not time limited. When participants reached the location that best approximated the target location, they pressed a button to indicate the end of the trial. The first type of test trial (Fig. 3, top) assessed spatial learning and memory relative to their own position (egocentric navigation): landmarks were removed, and participants navigated through the maze with the planks. Therefore, participants had to remember the path distance and direction taken through the maze to reach the target location. Unfortunately, the data from the first test could not be used because of a programming failure. There was a time gap between the end of the last training trial and the beginning of the test trial which meant that any head movements would change the facing direction at the starting location. Since the first test contained no landmarks, participants had no reference to determine which direction they were facing at the start of the trial. Therefore, only data from the second test trial are reported. The second test trial (Fig. 3, bottom) assessed spatial learning and memory relative to the landmarks: the planks were removed, and participants navigated through the pool with the surrounding landmarks (allocentric navigation). Therefore, participants had to remember the relative distance and direction from the landmarks to triangulate the target location.

Following the VR task, participants completed the DFS questionnaire and the backward digit span task. Experimental data are available at https://osf.io/mkptq/.

## Results

### Training data

**Fig. 4:**
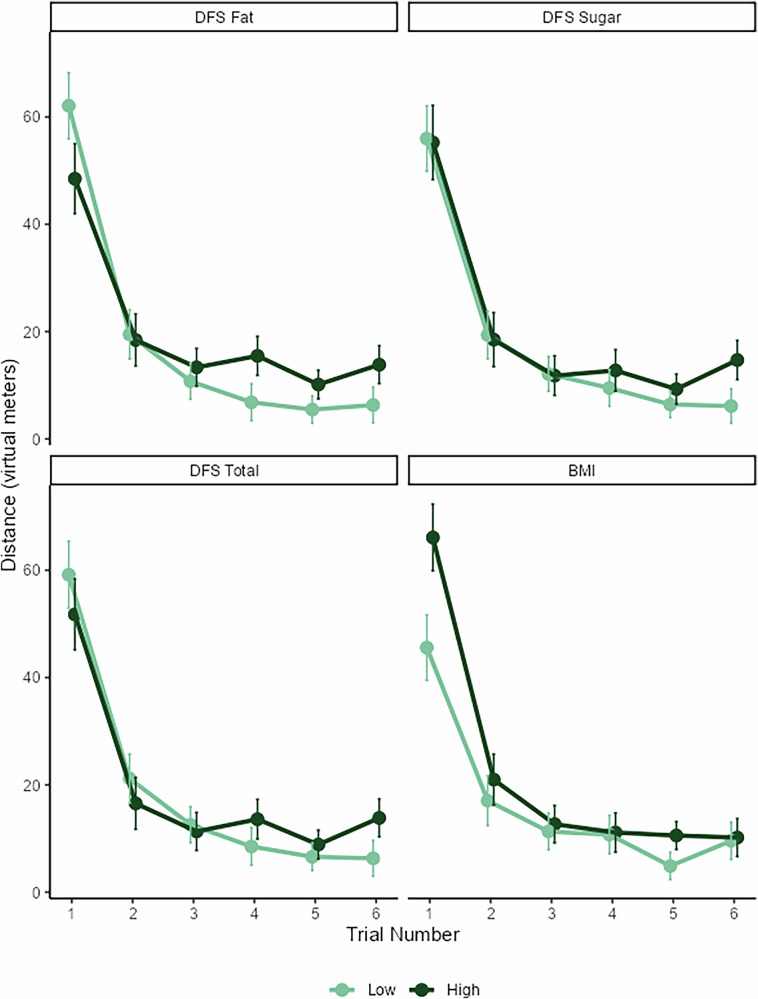
Performance as a function of trial number and diet. Performance is represented as the distance from the goal at the end of each trial. Smaller scores indicate better performance. Diet scores are median split. Error bars represent one standard error of the mean. DFS dietary fat and free sugar questionnaire. BMI body mass index.

Multilevel regression models used four health-related variables (DFS fat, DFS sugar, DFS total, and BMI; in separate models) and trial number to predict participants’ distance from the target at the conclusion of each of the training trials. The results of all models (see Supplementary Table 2) showed a main effect of trial number, with distance from the target decreasing across trials. There were no main effects of any of the diet variables, although DFS fat and DFS sugar approached significance, with higher diet scores trending towards a greater mean distance from the target. There were, however, significant interactions between the health variables and trial number, with DFS fat, DFS sugar, and DFS total moderating the trial effect, such that participants with higher sugar, fat, and total DFS scores showed flatter learning curves across the training. BMI did not significantly moderate the trial effect. These results are represented in Fig. 4, using a median split for high vs. low diet scores.

### Test data

Descriptive statistics and correlations are presented in Table 1. Test data are reported for the second test trial with the planks removed and the pool surrounded by landmarks (allocentric navigation). Both DFS fat (*r* = 0.28, *p* = 0.039) and DFS total (*r* = 0.29, *p* = 0.031) were significantly positively related to distance from the target at test, indicating that those with higher DFS fat and DFS total scores finished further from the target location (see Table 1). After controlling for BMI and working memory (Table 2), DFS total remained a significant predictor of test performance (β = 0.30, *p* = 0.030) but DFS Fat was now marginally non-significant (β = 0.27 *p* = 0.052).

**Table 1:** Means, standard deviations, and correlations for test performance and health-related variables.

	M (SD)	1	2	3	4
1. Test performance	24.09 (19.3)				
2. DFS fat	28.64 (5.92)	0.279*			
3. DFS sugar	12.29 (4.33)	0.223	0.507***	—	
4. DFS total	59.91 (12.82)	0.291*	0.837***	0.83***	—
5. BMI	23.06 (3.71)	−0.091	0.045	0.118	0.055

Test performance represents spatial memory ability, expressed as a distance score from the target location in arbitrary units. Smaller scores indicate better performance. DFS dietary fat and free sugar questionnaire. BMI body mass index. DFS fat is scored out of 60, DFS sugar is scored out of 30, and DFS total is scored out of 130. M and SD represent mean and standard deviation, respectively *p < 0.05, **p < 0.01, ***p < 0.001.

**Table 2:** Multilevel regression models of test data.

	distance					distance					distance				
*Predictors*	*Estimates*	*std. Beta*	*CI*	*standardized CI*	*p*	*Estimates*	*std. Beta*	*CI*	*standardized CI*	*p*	*Estimates*	*std. Beta*	*CI*	*standardized CI*	*p*
(Intercept)	−1.93	−0.00	−27.15 – 23.30	−0.26 – 0.26	0.879	11.87	−0.00	−3.72 – 27.46	−0.27 – 0.27	0.133	−2.19	−0.00	−26.49 – 22.12	−0.26 – 0.26	0.857
DFS fat	0.91	0.28	0.05 – 1.77	0.01 – 0.54	0.039										
DFS sugar						0.99	0.22	−0.20 – 2.19	−0.05 – 0.49	0.102					
DFS total											0.44	0.29	0.04 – 0.84	0.03 – 0.55	0.031
BMI															
B/ward digit span															
Observations	55					55					55				
R^2^ / R^2^ adjusted	0.078 / 0.060					0.050 / 0.032					0.085 / 0.068				
(Intercept)	2.17	0.00	−43.34 – 47.68	−0.27 – 0.27	0.924	20.37	0.00	−18.78 – 59.51	−0.27 – 0.27	0.301	2.43	0.00	−41.21 – 46.08	−0.27 – 0.27	0.911
DFS fat	0.94	0.27	−0.01 – 1.89	−0.00 – 0.55	0.052										
DFS sugar						1.15	0.25	−0.20 – 2.49	−0.04 – 0.53	0.092					
DFS total											0.48	0.30	0.05 – 0.91	0.03 – 0.58	0.030
BMI	−0.60	−0.11	−2.03 – 0.84	−0.39 – 0.16	0.409	−0.72	−0.14	−2.18 – 0.74	−0.42 – 0.14	0.326	−0.63	−0.12	−2.05 – 0.79	−0.39 – 0.15	0.376
B/ward digit span	1.50	0.13	−1.62 – 4.61	−0.14 – 0.41	0.339	1.13	0.10	−2.11 – 4.36	−0.19 – 0.39	0.487	1.30	0.12	−1.80 – 4.40	−0.16 – 0.39	0.403
Observations	52					52					52				
R^2^ / R^2^ adjusted	0.111 / 0.055					0.093 / 0.036					0.128 / 0.073				

## Discussion

This study used a virtual reality environment to examine the relationship between diet and spatial learning in people. We found that participants who reported higher scores on the DFS questionnaire, indicating greater consumption of HFHS foods, had flatter learning curves during training relative to those who reported lower DFS scores. Moreover, participants with higher scores were also worse at remembering the location of the target (the treasure chest) at test, when the plank system that had been used as a path through the maze was removed, leaving only the surrounding landmarks. This relationship remained even after controlling for BMI and working memory performance.

Evidence that consumption of HFHS diets impairs spatial learning in rodents suggested that similar effects would be detected in people [10, 18]. However, the training data failed to detect the main effect of diet (fat, sugar, or total scores) on spatial learning averaged across training trials. Nevertheless, there was a significant interaction such that participants with higher DFS scores (on fat, sugar, and total) showed flatter learning curves than those with lower scores. It needs to be noted that this interaction may be driven, in part, by differences in performance on trial 1, with those scoring higher on the DFS questionnaire starting with slightly lower values. Therefore, the interaction does not necessarily show that participants who consume more fats and sugars are slower to learn about spatial locations. A more compelling pattern of data for such a conclusion would be where performance was equivalent on trial 1 across dietary intake but diverging on subsequent training trials.

The test data provided stronger evidence for a negative association between intake of HFHS foods and performance in the spatial learning task, even after controlling for BMI and general executive functioning as indexed by a working memory task. Notably, performing the allocentric test with the planks removed but with the landmarks in place required participants to use distance and direction information to estimate the location of the target relative to the landmarks. The most precise estimation method would be to triangulate the target position relative to three landmarks encoded across training, but a less accurate method would be to use one or two landmark(s). The finding that participants with higher DFS fat and DFS total scores were further from the target location is in line with the finding that rats fed a HFHS diet are impaired in the use of distance and direction information (spatial geometry) to remember the location of objects in a rectangular arena [15].

Moreover, the current results also mirror those from rodent studies in two other important aspects. First, the spatial navigation deficit was present after controlling for BMI, suggesting that impairments are related to HFHS intake rather than weight gain per se. In rodent studies, HFHS diet-induced spatial deficits have been shown to emerge rapidly, after a few days and in advance of significant differences in body weight [e.g. [15, 25]], These results suggest that while consumption of HFHS diets leads to weight gain, cognitive deficits are the result of dietary exposure, and weight gain is not a necessary condition. However, the current sample had a restricted BMI range compared to the general population. It would provide additional support for the present findings were they replicated in a sample with a larger BMI range. Second, the spatial navigation deficit was present after controlling for working memory ability. In rodent studies, HFHS diet-induced cognitive deficits have been shown to selectively impair spatial learning and memory while leaving other forms of memory intact, such as object memory [8, 15] and recognition memory [14]. These results suggest that any deleterious effects of HFHS diets on brain morphology and cognitive function are selective, likely starting in the hippocampus, rather than acting globally across the brain.

More generally, the present results are consistent with a recent meta-analysis of human studies which found a small but significant impact of HFHS diets on hippocampal-dependent tasks [18]. However, past studies investigating the impact of diet on cognitive function have differed in the tasks used to assess learning and memory, e.g., Hopkins Verbal Learning Task [26, 27] and the Groton Maze Learning Test [5]. Although both tasks are thought to rely on the hippocampus, their sensitivity to diet-induced impairments is likely to differ. Indeed, there are animal and human studies that have failed to find an effect of diet on cognitive function [see [10, 18]]. Inconsistencies in the literature may be due to methodological differences in the assessment tasks selected, as well as the diet manipulations used in experimental studies and the diet reported in correlational studies. Tasks, such as that used here, which are modeled on those used in rodent studies that have revealed clear effects of diet on spatial learning, may be best suited to reveal such effects in people.

It should be acknowledged that the current study did not manipulate dietary intake, and the results are thus correlational in nature. Based on the findings from the animal and human literature, we have interpreted these findings to mean that poor dietary intake is associated with worse spatial navigation performance. Although less plausible, it is possible that the results may be driven by the opposite causal structure, specifically, that participants with worse spatial navigation ability are more attracted to foods high in fat and sugar and consume more of them. To our knowledge, no studies have directly manipulated or tested this possibility. The closest evidence for such a hypothesis is that rats with induced brain injury to the amygdala and hippocampus show impaired neophobia to foods [28]. Injured animals were more willing to try novel foods and distributed their eating evenly among different foods, while control animals preferred familiar foods. However, extending this finding to explain an attraction to HFHS foods among those with impaired spatial ability still requires a few assumptions to be met. For example, that HFHS foods are novel until the time of the brain injury or accruement of the spatial/hippocampal impairment, which seems unlikely.

Instead, we address the alternative explanation of the correlation between diet and spatial performance with two points: First, while the causal direction of spatial navigation impacting diet seems unlikely given the evidence from rodent studies [e.g. [10]], both may be caused by an underlying common cause, e.g., impaired hippocampal function. For example, Kanoski and Davidson [29] have proposed a model of dietary intake whereby HFHS consumption impairs hippocampal-dependent functions, including learning, memory, and appetite control; impairments which, in turn, drive and exacerbate consumption of HFHS foods, leading to a “vicious cycle” [see also [27, 30–32]]. However, although this proposal supposes a feedback loop such that diet impairs hippocampal-dependent processes which in turn promotes further intake of the diet, it nevertheless assumes that the critical factor is the effect of the diet on hippocampal-dependent cognition, such as the use of spatial information in navigation. Second, the rodent literature strongly supports the causal direction we have proposed. In addition to experimental manipulations that assess spatial learning and memory following exposure to HFHS diets compared to control diets, other forms of experimental manipulations also support the direction that HFHS diets impair spatial navigation ability. For example, studies that have removed the HFHS diets show that spatial impairments can be reversed [13, 33]. Further, studies have also shown that spatial learning prior to HFHS diet exposure is protected and does not suffer impairment [13]. These manipulations provide compelling evidence that dietary consumption of HFHS diets is responsible for spatial learning and memory impairments in rodents. It would be worthwhile conducting similar experimental studies in humans using a diet reversal and pre-diet learning design to test the causal direction of the findings reported here. The benefit of such designs is that they aim to improve any existing spatial impairments through a reversal from a HFHS to a healthy diet switch or show that existing spatial memories are protected from healthy to HFHS diet switch.

As far as we are aware, the present study is the first to examine the effects of diet on “true” spatial navigation ability in people, true in the sense of explicitly requiring participants to learn and remember a route using distance and direction information at eye level. Previous studies have used non-spatial hippocampal-dependent tasks (e.g., the Hopkins Verbal Learning Task) or maze learning tasks that do not require spatial learning and memory. For example, in the Groton Maze Learning Test, participants are required to learn and remember a path within a grid from a starting square to the target square. This type of task assesses short-term memory and/or visual working memory. Similarly, any maze task that requires remembering a path from a top-down view does not exclusively involve spatial navigation ability. Tasks that involve remembering the location of objects in a scene or a specific location on a grid, are closer to assessing spatial ability but can be solved using alternative strategies that do not rely on spatial information (e.g., relational memory). In contrast, the virtual reality environment required participants to learn a path through the maze at eye level (or “street view”; c.f. top-down view) and remember the route using distance and direction information relative to the self or relative to the surrounding landmarks, processes used by people to navigate and remember spatial routes in their physical or normal environment. However, using virtual reality to assess spatial ability has some drawbacks, particularly the attrition rate due to motion sickness. Matching the movements and distances in physical space with the movements and distances in virtual space would be a way to reduce the motion sickness induced by misalignments with the vestibular system. Advancements in technology are likely to improve the optic flow of visual information and reduce motion sickness, making VR a promising tool for studying spatial navigation in the future.

In summary, the current experiment showed that consumption of HFHS foods, measured using a dietary questionnaire estimating types of food consumed over a 12-month period, predicted performance in a spatial memory test assessed using virtual reality. Young adults who reported consuming more HFHS foods across that period were worse at remembering the target location in the maze. This relationship remained even after controlling for BMI and working memory ability, suggesting that the impairments are related to dietary consumption, above and beyond body weight; and are not general to executive functioning ability. These results are consistent with the growing animal literature on the impact of diet on spatial learning and memory. They also suggest that the hippocampus—especially the regions important for spatial ability—is particularly sensitive to the deleterious effects of a HFHS diet. To our knowledge, the current study is the first to link the type of diet consumed with true spatial ability in people, requiring participants to learn and remember a route using distance and direction information at eye level. The findings affirm the importance of healthy dietary choices for cognitive well-being.

## Supplementary information

Supplementary material

## Data Availability

Primary data are publicly available https://osf.io/mkptq/.
