# Supplementary material for: Consumption of a diet high in fat and sugar is associated with worse spatial navigation ability in a virtual environment
Source: Int J Obes (Lond). 2025 Apr 17;49(7):1354–62. doi: 10.1038/s41366-025-01776-8 (PMC12283396; doi:10.1038/s41366-025-01776-8)
Supplement: Supplementary file 1 — Supplementary material [file 41366_2025_1776_MOESM1_ESM.docx]

Supplementary Table 1. Demographic breakdown of participants who did and did not complete the VR task.

|  | **VR data complete** | | | **VR data incomplete** | | |
| --- | --- | --- | --- | --- | --- | --- |
|  | n | Mean | SD | n | Mean | SD |
| Age | 55 | 19.95 | 3.2 | 46 | 20.39 | 4.31 |
| Height | 55 | 172.78 | 9.63 | 45 | 168.22 | 7.74 |
| Weight | 55 | 69.15 | 13.72 | 45 | 60.64 | 10.87 |
| BMI | 55 | 23.06 | 3.71 | 45 | 21.39 | 3.35 |
| DFS fat | 55 | 28.64 | 5.92 | 46 | 28.57 | 5.53 |
| DFS sugar | 55 | 12.29 | 4.33 | 46 | 12.11 | 3.37 |
| DFS total | 55 | 59.91 | 12.82 | 46 | 59.5 | 10.52 |
| B/ward digit span | 52 | 6.1 | 1.74 | 39 | 6.51 | 2.34 |
|  |  |  |  |  |  |  |
| One participant with complete VR data, and one participant with incomplete VR data did not report their gender. Female participants had a higher dropout rate (56%) compared to male participants (32%). This suggests that female participants were more likely to experience motion sickness in our VR task. The differences in Height, Weight, and BMI can be attributed to the differences in dropout rates between females and males. | | | | | | |

Supplementary Table 2. Multilevel regression models of training data.

|  | **distance** | | | | | | **distance** | | | | | | **distance** | | | | | | **distance** | | | | | |
| --- | --- | --- | --- | --- | --- | --- | --- | --- | --- | --- | --- | --- | --- | --- | --- | --- | --- | --- | --- | --- | --- | --- | --- | --- |
| *Predictors* | *Estimates* | *std. Beta* | *CI* | *standardized CI* | *p* | *std. p* | *Estimates* | *std. Beta* | *CI* | *standardized CI* | *p* | *std. p* | *Estimates* | *std. Beta* | *CI* | *standardized CI* | *p* | *std. p* | *Estimates* | *std. Beta* | *CI* | *standardized CI* | *p* | *std. p* |
| (Intercept) | 71.54 | 0.00 | 41.40 – 101.69 | -0.11 – 0.11 | **<0.001** | 1.000 | 61.80 | 0.00 | 43.49 – 80.11 | -0.11 – 0.11 | **<0.001** | 1.000 | 66.86 | 0.00 | 37.81 – 95.91 | -0.11 – 0.11 | **<0.001** | 1.000 | 29.47 | 0.00 | -9.14 – 68.09 | -0.11 – 0.11 | 0.134 | 1.000 |
| trial | -15.13 | -0.47 | -22.55 – -7.71 | -0.56 – -0.38 | **<0.001** | **<0.001** | -12.76 | -0.47 | -17.27 – -8.25 | -0.56 – -0.38 | **<0.001** | **<0.001** | -15.70 | -0.47 | -22.86 – -8.53 | -0.56 – -0.38 | **<0.001** | **<0.001** | -4.12 | -0.47 | -13.65 – 5.41 | -0.56 – -0.37 | 0.396 | **<0.001** |
| DFS fat | -0.91 | 0.00 | -1.94 – 0.12 | -0.11 – 0.12 | 0.085 | 0.938 |  |  |  |  |  |  |  |  |  |  |  |  |  |  |  |  |  |  |
| trial × DFS fat | 0.26 | 0.10 | 0.01 – 0.52 | 0.00 – 0.19 | **0.041** | **0.041** |  |  |  |  |  |  |  |  |  |  |  |  |  |  |  |  |  |  |
| DFS sugar |  |  |  |  |  |  | -1.32 | 0.03 | -2.73 – 0.09 | -0.09 – 0.14 | 0.066 | 0.648 |  |  |  |  |  |  |  |  |  |  |  |  |
| trial × DFS sugar |  |  |  |  |  |  | 0.42 | 0.11 | 0.08 – 0.77 | 0.02 – 0.21 | **0.016** | **0.016** |  |  |  |  |  |  |  |  |  |  |  |  |
| DFS total |  |  |  |  |  |  |  |  |  |  |  |  | -0.36 | 0.06 | -0.83 – 0.12 | -0.05 – 0.17 | 0.142 | 0.320 |  |  |  |  |  |  |
| trial × DFS total |  |  |  |  |  |  |  |  |  |  |  |  | 0.14 | 0.11 | 0.02 – 0.25 | 0.02 – 0.20 | **0.023** | **0.023** |  |  |  |  |  |  |
| BMI |  |  |  |  |  |  |  |  |  |  |  |  |  |  |  |  |  |  | 0.70 | 0.02 | -0.96 – 2.35 | -0.09 – 0.14 | 0.407 | 0.674 |
| trial × BMI |  |  |  |  |  |  |  |  |  |  |  |  |  |  |  |  |  |  | -0.15 | -0.03 | -0.56 – 0.26 | -0.13 – 0.06 | 0.475 | 0.475 |
| **Random Effects** | | | | | | | | | | | | | | | | | | | | | | | | |
| σ^2^ | 551.40 | | | | | | 548.23 | | | | | | 549.37 | | | | | | 558.86 | | | | | |
| τ_00_ | 41.71 _ID_ | | | | | | 41.73 _ID_ | | | | | | 39.60 _ID_ | | | | | | 40.04 _ID_ | | | | | |
| ICC | 0.07 | | | | | | 0.07 | | | | | | 0.07 | | | | | | 0.07 | | | | | |
| N | 55 _ID_ | | | | | | 55 _ID_ | | | | | | 55 _ID_ | | | | | | 55 _ID_ | | | | | |
| Observations | 330 | | | | | | 330 | | | | | | 330 | | | | | | 330 | | | | | |
| Marginal R^2^ / Conditional R |  |  |  |  |  |  |  |  |  |  |  |  |  |  |  |  |  |  |  |  |  |  |  |  |
